# Removal of a Needle from the Heart

**Published:** 1873-05

**Authors:** 


Removal of a Needle from the Heart.—In the session of the Royal Medical and Surgical Society of February the 1st, of this year, Callender reports the case of a man, into the apex of whose heart—by what occurrence is not stated—a sewing needle had entered. The patient attended to his business for nine days, although in pain all the time. On that day an incission was made into the fifth intercostal space, the broken end of the needle was discovered, and by traction removed from the heart. Patient recovered completely, without further sickness. Referant illustrates by drawings the motions which the broken end of the needle was seen to make after the incision into the intercostal space, corresponding completely with the motions of the heart. (This case speaks in justification of the proposition to use electro-puncture of the heart, made by Steiner in No. 22, of this journal, in chloroform asphyxia.)—*Berliner Klinische Wochenschrift, No.* 12, 1873.

